# Periodic Density Matrix Embedding for CO Adsorption on the MgO(001) Surface

**DOI:** 10.1021/acs.jpclett.2c01915

**Published:** 2022-08-08

**Authors:** Abhishek Mitra, Matthew R. Hermes, Minsik Cho, Valay Agarawal, Laura Gagliardi

**Affiliations:** † Department of Chemistry, Chicago Center for Theoretical Chemistry, University of Chicago, Chicago, Illinois 60637, United States; ‡ Department of Chemistry, Brown University, Providence, Rhode Island 02912, United States; § Department of Chemistry, Pritzker School of Molecular Engineering, James Franck Institute, Chicago Center for Theoretical Chemistry, University of Chicago, Chicago, Illinois 60637, United States; ∥ Argonne National Laboratory 9700 South Cass Avenue Lemont, Illinois 60439, United States

## Abstract

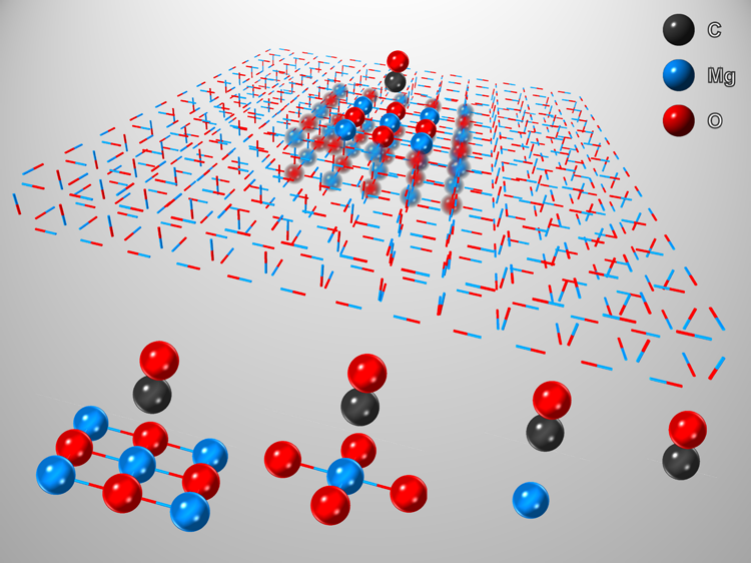

The adsorption of simple gas molecules to metal oxide surfaces is a primary step in many heterogeneous catalysis applications. Quantum chemical modeling of these reactions is a challenge in terms of both cost and accuracy, and quantum-embedding methods are promising, especially for localized chemical phenomena. In this work, we employ density matrix embedding theory (DMET) for periodic systems to calculate the adsorption energy of CO to the MgO(001) surface. Using coupled-cluster theory with single and double excitations and second-order Møller–Plesset perturbation theory as quantum chemical solvers, we perform calculations with embedding clusters up to 266 electrons in 306 orbitals, with the largest embedding models agreeing to within 1.2 kcal/mol of the non-embedding references. Moreover, we present a memory-efficient procedure of storing and manipulating electron repulsion integrals in the embedding space within the framework of periodic DMET.

Magnesium oxide (MgO) surface plays an important role in several heterogeneous catalytic reactions, such as the partial oxidation of methane,^1^ the Guerbet reaction at a low pressure,^2^ the synthesis of 2-amino-2-chromenes using benign reactants,^3^ the conversion of ethane to ethylene,^4^ and the formation of carbonates from carbon monoxide in the presence of oxygen.^5^ Modeling surface adsorption of simple molecules, for example, carbon monoxide (CO), to metal oxide surfaces, like MgO, is an important step for theorists toward the understanding of heterogeneous catalysis, but it is challenging.^6−9^ The CO molecule binds to the MgO surface, preferentially with a C–Mg interaction.^10^ The CO/MgO adsorption energy is relatively small, and a range of values has been obtained by different experimental techniques and theoretical methods. An adsorption energy of 3.23 kcal/mol was obtained from thermodesorption experiments by Wichtendahl et al.,^11^ whereas temperature-programmed desorption (TPD) experiments performed by Dohnálek et al.^12^ accounted for an adsorption energy of 4.84 kcal/mol. An experimental study by Xu et al. reported an interaction energy of 3.0 kcal/mol.^13^ For a more extensive description of the rich experimental history of the MgO/CO adsorption, readers are referred to the review by Spoto et al.^14^

Computationally, the challenge posed by this system is the weak interaction between CO and the surface, mainly arising from van der Waals (vDW) forces. Many local and semi-local Kohn–Sham density functionals^15,16^ are unable to account for vDW interactions in such cases.^17−20^ The accurate estimation of the adsorption energy, therefore, requires an extensive testing of density functional theory (DFT) functionals and the incorporation of dispersion corrections.^21−25^ On the other hand, size-consistent correlated wave function (CWF)-based *ab initio* methods can model vDW interactions,^17^ and, in the past few years, their application to periodic systems has gained momentum.^26−33^ An attractive feature of CWF methods is their systematic improvability; however, their steep computational cost scaling with system size poses an obstacle.^17,34^ This becomes apparent in applications where one cannot exploit translational symmetry as a result of the presence of irregularities in the crystal, like point defects or surface adsorbates.

Among the most recent wave function theoretical studies, Staemmler computed an adsorption energy of 2.86 kcal/mol using the method of local increments,^17^ whereas, using their combined MP2-CCSD(T) approach embedded in a potential of point charges, Boese et al.^8^ calculated an adsorption energy of 5.0 kcal/mol, but also pointed out the wide range of numbers that can be obtained using different electronic structure theories. Valero et al.^6^ showed that the Minnesota functionals M06-2X and M06-HF provide adsorption energies of around 6 kcal/mol.

These systems are usually modeled by either cutting a cluster from an extended system (cluster modeling) or assuming periodic boundary conditions (PBCs). Defining a cluster involves choosing an appropriate cluster size and saturating the free valencies using hydrogen atoms, which can create spurious electronic states at the boundary. Previously used cluster models^8,9,35−39^ were surrounded with point charges or periodic potentials to replicate the environment. On the other hand, modeling surface adsorption with PBCs using CWF becomes prohibitively costly as a result of the apparent need of large supercells (often hundreds of atoms). To overcome the cost and maintain the accuracy of the parent method, the models can be subjected to fragmentation/embedding approaches.^8,31,32,40,41^ Quantum embedding methods use a high-level quantum chemistry solver to represent a small region of interest (here referred to as the fragment/impurity), whereas the rest of the system (generally referred to as “environment”) is represented using a mean-field method, such as Kohn–Sham density functional theory (KS-DFT)^15,16^ or Hartree–Fock (HF).^34,42^ Modeling the adsorption of CO to a MgO surface is therefore ideal to investigate the performance of wave function-in-wave function quantum embedding approaches.

In this work, we use the density matrix embedding theory (DMET) algorithm to calculate the adsorption energy of a CO molecule to the MgO(001) surface. DMET, a wave function-in-wave function embedding technique,^43^ was originally proposed as a promising alternative to dynamical mean-field theory (DMFT)^44^ to treat strongly correlated fermions in the one-dimensional Hubbard model. Several theoretical developments and targeted applications have followed since.^45−54^ DMET uses the Schmidt decomposition^55^ of a mean-field wave function to model the environment of a given impurity space using an effective bath. Pham et al.^56^ and Cui et al.^57^ extended the DMET algorithm to periodic systems. Here, we use the coupled-cluster theory with single and double excitations (CCSD) and second-order Møller–Plesset perturbation theory (MP2) as high-level solvers within the DMET formalism and compare them to non-embedding Γ-point CCSD and MP2 references.

The DMET calculations are performed with the periodic DMET (pDMET) code,^58,59^ which uses the electron integrals and quantum chemical solvers from the PySCF package.^60,61^ Similar to the workflow in ref (62), we first perform a HF calculation to obtain the mean-field wave function. Next, we define the impurity region using a set of localized orbitals in real space. Here, we use the maximally localized Wannier functions (MLWFs),^63,64^ implemented in the wannier90^65^ code via the pyWannier90 interface.^66^ Because adsorbates (i.e., perturbations to the pristine crystal) are introduced, the Brillouin zone is sampled at the Γ point and a subset of the MLWFs (which we label as *N*_imp_) at the chemical region of interest, for example, those around the adsorbate, are chosen to define the impurity. The bath is defined using the Schmidt decomposition,^46^ where the environment block (*D*_env_) of the one-body reduced density matrix (1-RDM) is diagonalized as follows:
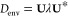
1where λ is a diagonal matrix of eigenvalues λ_*i*_ (*i* = 0, 1, ..., *N*_env_, where *N*_env_ is the number of the environment orbitals). The columns of the unitary matrix **U** corresponding to λ_*i*_ other than zero or two define the entangled bath orbitals; the remainder orbitals are treated as a frozen core in the embedding calculation. The mean-field wave function after the Schmidt decomposition thus has the following form:
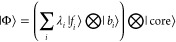
2where , , and  are single determinants in the Fock spaces of the *N*_imp_ impurity orbitals, *N*_bath_ ≤ *N*_imp_ bath orbitals, and *N*_core_ frozen core orbitals, respectively, and *i* = 0, 1, ..., *N*_imp_. The high-level wave function, , diagonalizes the embedding Hamiltonian, 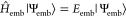
3where  is the partial trace of the Hamiltonian over the  determinant; its operator terms involve only *N*_emb_ = *N*_imp_ + *N*_bath_ ≤ 2*N*_imp_ embedding orbitals. For calculations of energy differences, it is important to choose the same number of *N*_emb_ embedding orbitals for the different geometries. As discussed later, the computational cost of the high-level method is thus reduced by not requiring to have *N*_core_ orbitals in the effective Hamiltonian.

We use a density fitting (DF) approach based on the Cholesky decomposition,^67−70^ where four-center electron repulsion integrals (ERIs) in the embedding space can be reconstructed in terms of the three-center ERIs as
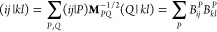
4where *P* and *Q* represent auxiliary basis functions, **M**_*PQ*_ = (*P* |*Q*) is the Coulomb metric, and  and  are the Cholesky vectors
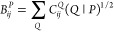
5where the expansion coefficients  are obtained by solving a linear equation
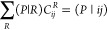
6The auxiliary basis set contains even-tempered basis (ETB) functions generated with a progression factor β = 2.0 for the auxiliary expansion of the polarized double-ζ basis set (GTH-DZVP) and polarized triple-ζ basis set (GTH-TZVP) bases and will be represented using *P* and *Q*. The prefix GTH is used because these basis sets are consistent with the Goedecker–Teter–Hutter pseudopotentials^71,72^ that have been used for all of the calculations. Our approach is agnostic to whether we use pseudopotentials or an all-electron basis sets because surface adsorption primarily depends upon valence electrons and orbitals.

In the previous implementations of periodic DMET,^56,57^ the quantum impurity solvers used four-center two-electron integrals obtained by contracting the three-center electron repulsion integrals (ERIs) in the embedding space as in eq 4. This eliminated the full-basis 4-index ERI array but still required the storage of the 4-index ERIs in the embedding basis, whose memory cost scales with the size of the impurity as .^56,57^ On the other hand, in the current implementation, we use the DF for the MP2 and CCSD high-level solvers, in which the programmable equations for the energy are implemented in terms of the Cholesky vectors themselves and the (*ij*|*kl*) integrals in the embedding basis are not required. In other words, the right-hand side of eq 4 is not evaluated but is algebraically substituted into the energy expressions in the high-level solver implementations. The formal memory cost scaling of this approach (with respect to the size of the impurity) is , which, in practice, is much more favorable for our applications, as shown later.

We compute the adsorption energy, Δ*E*, as the difference between the energy at the equilibrium geometry, *E*_eq_ (C–Mg bond distance of 2.479 Å),^8^ and at a separated geometry, *E*_sep_ (C–Mg bond distance of 6 Å), as indicated in Figure 1a. The 4 × 4 × 2 slab model of MgO has a vacuum of approximately 16 Å in the vertical direction (collinear to CO) above the MgO surface to avoid the interaction between neighboring images. We consider four choices of impurity clusters, as shown in Figure 1b. For these four choices, the orbitals are localized on (a) only the CO molecule, (b) the CO molecule and the nearest Mg atom on the substrate (denoted as CO + Mg), (c) the CO molecule, the nearest Mg atom, and the 4 nearest O atoms on the substrate (denoted as CO + MgO_4_), and finally (d) the 4 next to nearest Mg atoms in addition to choice (c) (denoted as CO + Mg_5_O_4_). The orbitals localized at the highlighted atoms in the embedding clusters (Figure 1b) have been considered as the fragment. We do not correct for basis set superposition error (BSSE) in our calculations, because this would require the use of ghost basis functions. The Schmidt decomposition is unable to produce bath orbitals entangled to the unoccupied ghost orbitals; therefore, these correction calculations would be systematically deficient in bath orbitals compared to the calculation being corrected. A proper way to account for the most entangled orbitals from the environment is desired especially for physical/chemical phenomena, where BSSE is non-negligible and is currently an area upon which we are working.

**Figure 1:**
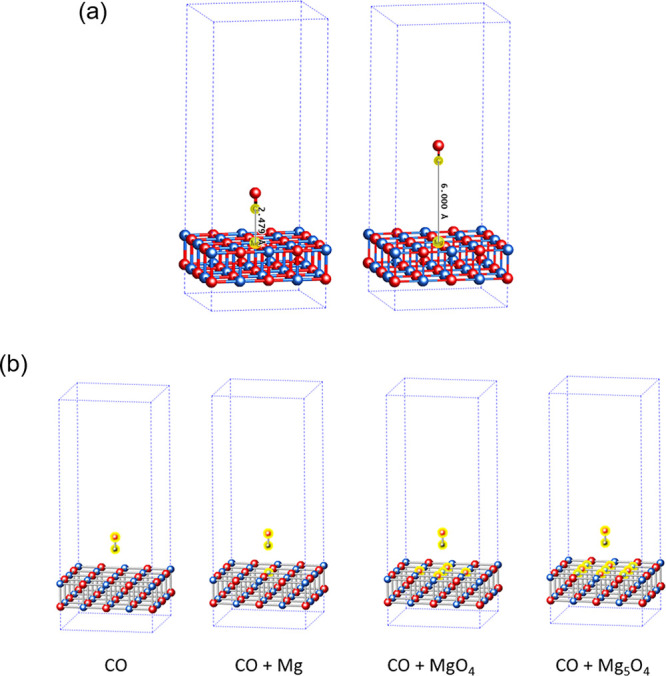
(a) CO at a distance of 2.479 Å (left) representing the geometry at equilibrium (referred as eq) and 6 Å (referred as sep) from the MgO surface (right) representing the geometry when there is no interaction between the substrate and adsorbate. Magnesium (Mg) atoms are shown in red; oxygen (O) atoms are shown in blue; and carbon (C) atoms are shown in gray. (b) Atoms highlighted in yellow form the impurity clusters used for DMET calculations.

In Figure 2, we report the relative energy *E*_rel_ of the CO + MgO model as a function of the distance between C (in CO) and Mg (in MgO) from 2 to 6 Å. We take as a reference value the total energy at the C–Mg distance of 2.479 Å, and *E*_rel_ at all other geometries are reported relative to this reference. The results are obtained using periodic MP2 calculations, restricted HF (RHF), and DMET-MP2. The DMET-MP2 calculations are performed using the smaller CO + Mg impurity subspace and the larger CO + MgO_4_ impurity subspace.

**Figure 2:**
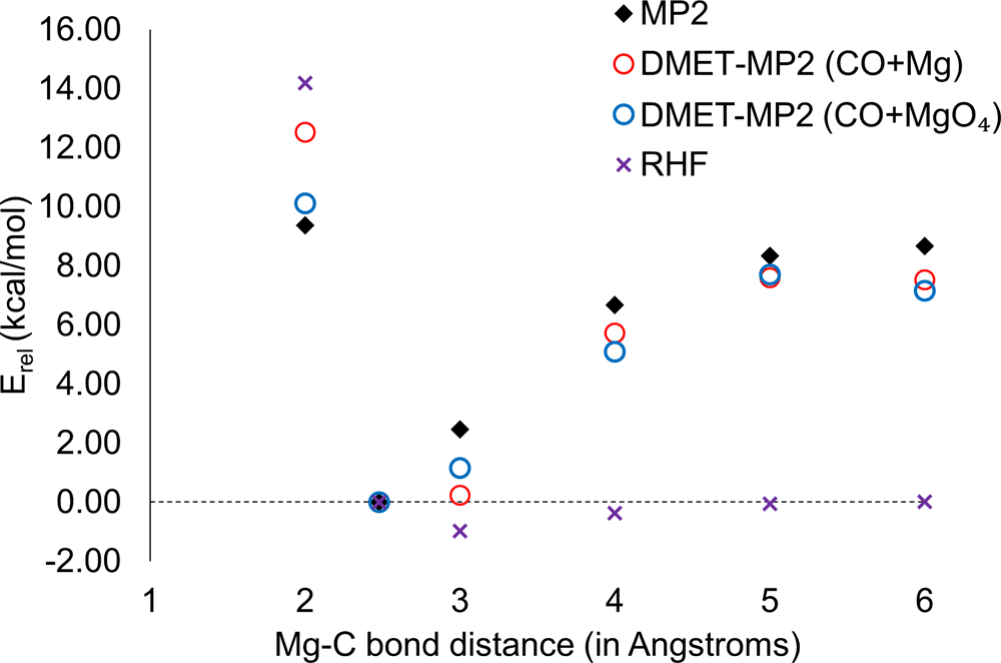
Relative energies *E*_rel_ (kcal/mol) obtained using non-embedding MP2 (blue diamond), DMET-MP2 with the CO + Mg impurity cluster (red circles), DMET-MP2 with the CO + MgO_4_ impurity cluster (dark blue circles) and RHF (purple crosses). The abscissa represents the Mg–C distances in angstroms. All *E*_rel_ values are reported as differences with respect to the value at the C–Mg distance of 2.479 Å. All calculations are performed using the DZVP basis set.

For the MP2 reference method, *E*_rel_ reaches an asymptotic value at a C–Mg distance of 6 Å and differs from *E*_rel_ at 5 Å by only 0.3 kcal/mol, thereby suggesting that 6 Å is a reasonable choice for a separated geometry. Using the CO + MgO_4_ fragment, the DMET *E*_rel_ values at each geometry are within 2 kcal/mol of the MP2 references. Using the CO + Mg fragment, the DMET value at the C–Mg bond distance of 2 Å has a large disagreement (ca. 5 kcal/mol) with the non-embedding reference, suggesting the importance of using a larger fragment space. The RHF *E*_rel_ values deviate significantly from the MP2 references. Morover, the RHF *E*_rel_ values at 3, 4, and 5 Å are negative, thereby indicating the presence of a minimum C–Mg bond length significantly away from the literature value of 2.479 Å.^8^ This is consistent with cluster HF calculations by Nygren et al.^73^ DMET on the other hand reproduces the binding energy (to within 1.5 kcal/mol) that is predicted by the reference.

Next, DMET calculations with the embedding clusters are compared to the periodic Γ-point CCSD and MP2 calculations (termed as the non-embedding references). The energy differences Δ*E* calculated using DMET-CCSD and DMET-MP2 with different basis sets are shown in Figure 3. The numbers are reported in Tables S2 and S3 of the Supporting Information. Four different basis set compositions have been used. They are divided as either DZVP on all of the atoms or TZVP on important atoms and DZVP on all of the others. TZVP (X) refers to TZVP applied on the X set of atoms and DZVP on all others.

**Figure 3:**
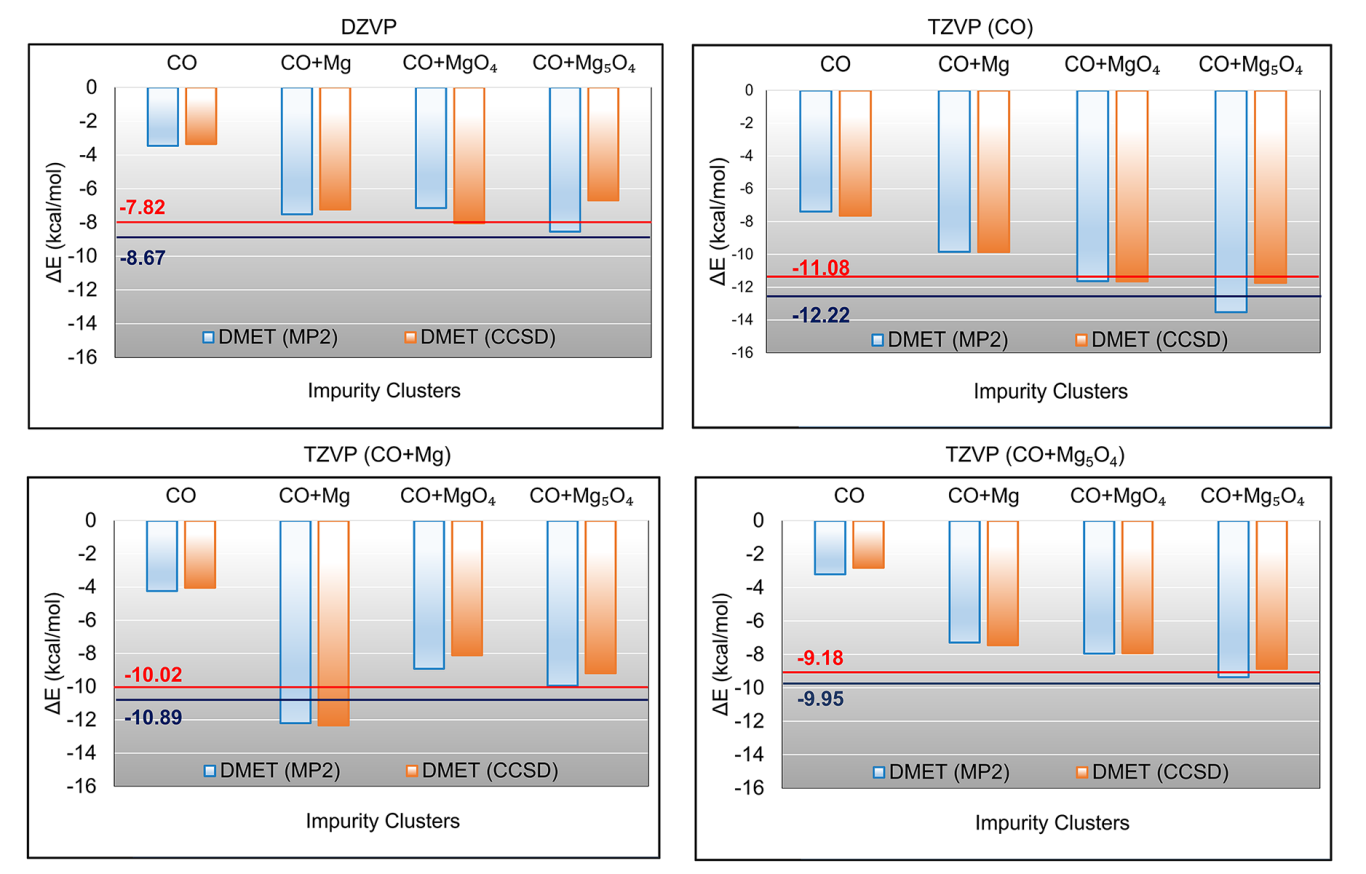
Adsorption energies (Δ*E*) between the equilibrium (2.479 Å) and separated (6 Å) geometries calculated using different basis sets and impurity cluster models. TZVP (X) refers to X being treated with the TZVP basis set and the rest of the system using the DZVP basis set. The solid lines correspond to the periodic Γ-point CCSD/MP2 calculations with red/dark blue color coding, respectively.

With the two largest impurity clusters, there is a closer agreement with the non-embedding references. This suggests that a larger number of surface atoms in the DMET impurity space is necessary for better accuracy. In Figure S1 of the Supporting Information, we plot the mean absolute deviations (MADs) from the non-embedding references and report them in Table S3 of the Supporting Information.

The requirement of bigger impurity clusters implies the storage and manipulation of a higher number of electron repulsion integrals (ERIs). With our DF implementation, we observe a severe reduction in memory requirements. For the test case of the CO + Mg_5_O_4_ fragment at the equilibrium geometry with more than 200 orbitals, the 4c–2e calculation requires 200 Gb of memory on a AMD EPYC 7502 32-core processor, while the DF integral calculation requires 30 Gb of memory. This is because the previous implementation in refs (56 and 62) required storage of  two-electron integrals, whereas the new implementation requires storage of  decomposed intermediates, where *N*_imp_ is the number of impurity fragment orbitals and *N*_aux_ is the number of auxiliary density-fitting orbitals. For large impurity fragments,  and the storage saving becomes significant. All of the calculations use multithreading (i.e., shared memory) only; there is no multiprocessing. It should be noted that Cui et al.^57^ also mentioned the possible memory efficiency that can be achieved by incorporating a similar algorithm. The current algorithm does not involve a formal speedup in terms of time required for a particular calculation. In this particular example, the 4c–2e calculation requires a wall time of 2 h and 21 mins, whereas the DF integral calculation requires a wall time of 1 h and 55 min.

Now, we examine the scaling of CCSD-DMET compared to the reference CCSD calculations. The computational cost of DMET is mainly dominated by the cost of the CCSD calculations within the embedding space. The most expensive term required in CCSD has a scaling of , where *N*_vir_ is the number of virtual orbitals and *N*_occ_ is the number of occupied orbitals.^42^ The primary contribution to the large scaling arises from the virtual orbitals. In the current framework, most of the virtual orbitals are part of the environment (i.e., a part of *N*_core_ orbitals), thereby significantly reducing the cost. If all *N*_core_ unentangled orbitals are doubly occupied, the scaling of CCSD-DMET becomes ; if they are all empty, the scaling of CCSD-DMET becomes .

In summary, we have used a periodic implementation of DMET to calculate the adsorption energy of the CO molecule with the MgO(001) surface. We have investigated two widely used quantum chemical solvers, CCSD and MP2, as high-level methods. We infer that DMET-CCSD and DMET-MP2 can be used to obtain adsorption energies with high accuracy and at a significantly lower cost compared to the non-embedding references. We additionally observed that an impurity cluster including at least a MgO_4_ moiety on the MgO surface is required for accurate adsorption energies. Therefore, we implemented an efficient way to store and manipulate the memory-intensive ERIs within the periodic DMET algorithm. We envision that, with our recent implementation of the multireference solvers,^62^ such as complete active space self-consistent field (CASSCF)^74−76^ and *n*-electron valence state second-order perturbation theory (NEVPT2),^77−80^ this approach can allow us to study bond-breaking phenomena of multireference systems on surfaces, at an affordable cost, which would be otherwise non-trivial for mean-field and single reference methods.

## Supporting Information Available

Mean absolute deviations (MADs), adsorption energies, relative energies for C–Mg distances, memory savings for DF integrals, sample input and output files, and total energies (PDF)
Transparent Peer Review report available (PDF)

## References

[ref1] Ito T.; Wang J. X.; Lin C. H.; Lunsford J. H. Oxidative Dimerization of Methane over a Lithium-Promoted Magnesium Oxide Catalyst. J. Am. Chem. Soc. 1985, 107, 5062–5068. 10.1021/ja00304a008.

[ref2] Ueda W.; Kuwabara T.; Ohshida T.; Morikawa Y. A low-pressure guerbet reaction over magnesium oxide catalyst. J. Chem. Soc., Chem. Commun. 1990, 1558–1559. 10.1039/c39900001558.

[ref3] Kumar D.; Reddy V. B.; Mishra B. G.; Rana R.; Nadagouda M. N.; Varma R. S. Nanosized magnesium oxide as catalyst for the rapid and green synthesis of substituted 2-amino-2-chromenes. Tetrahedron 2007, 63, 3093–3097. 10.1016/j.tet.2007.02.019.

[ref4] Morales E.; Lunsford J. H. Oxidative dehydrogenation of ethane over a lithium-promoted magnesium oxide catalyst. J. Catal. 1989, 118, 255–265. 10.1016/0021-9517(89)90315-1.

[ref5] Smart R. S. C.; Slager T. L.; Little L. H.; Greenler R. G. Carbon monoxide adsorption on magnesium oxide. J. Phys. Chem. 1973, 77, 1019–1023. 10.1021/j100627a011.

[ref6] Valero R.; Gomes J. R. B.; Truhlar D. G.; Illas F. Good performance of the M06 family of hybrid meta generalized gradient approximation density functionals on a difficult case: CO adsorption on MgO(001). J. Chem. Phys. 2008, 129, 124710 10.1063/1.2982923. 19045051

[ref7] Valero R.; Gomes J. R. B.; Truhlar D. G.; Illas F. Density functional study of CO and NO adsorption on Ni-doped MgO(100). J. Chem. Phys. 2010, 132, 104701 10.1063/1.3340506. 20232978

[ref8] Boese A. D.; Sauer J. Accurate adsorption energies of small molecules on oxide surfaces: CO-MgO(001). Phys. Chem. Chem. Phys. 2013, 15, 16481–16493. 10.1039/c3cp52321g. 23949344

[ref9] Alessio M.; Usvyat D.; Sauer J. Chemically Accurate Adsorption Energies: CO and H_2_O on the MgO(001) Surface. J. Chem. Theory Comput. 2019, 15, 1329–1344. 10.1021/acs.jctc.8b01122. 30596490

[ref10] Platero E. E.; Scarano D.; Spoto G.; Zecchina A. Dipole coupling and chemical shifts of CO and NO adsorbed on oxides and halides with rock-salt structure. Faraday Discuss. 1985, 80, 183–193. 10.1039/dc9858000183.

[ref11] Wichtendahl R.; Rodriguez-Rodrigo M.; Härtel U.; Kuhlenbeck H.; Freund H.-J. Thermodesorption of CO and NO from Vacuum-Cleaved NiO(100) and MgO(100). phys. stat. sol. (a) 1999, 173, 93–100. 10.1002/(SICI)1521-396X(199905)173:1<93::AID-PSSA93>3.0.CO;2-4.

[ref12] Dohnálek Z.; Kimmel G. A.; Joyce S. A.; Ayotte P.; Smith R. S.; Kay B. D. Physisorption of CO on the MgO(100) Surface. J. Phys. Chem. B 2001, 105, 3747–3751. 10.1021/jp003174b.

[ref13] Xu Y.; Li J.; Zhang Y.; Chen W. CO adsorption on MgO(001) surface with oxygen vacancy and its low-coordinated surface sites: Embedded cluster model density functional study employing charge self-consistent technique. Surf. Sci. 2003, 525, 13–23. 10.1016/S0039-6028(02)02566-9.

[ref14] Spoto G.; Gribov E. N.; Ricchiardi G.; Damin A.; Scarano D.; Bordiga S.; Lamberti C.; Zecchina A. Carbon monoxide MgO from dispersed solids to single crystals: A review and new advances. Prog. Surf. Sci. 2004, 76, 71–146. 10.1016/j.progsurf.2004.05.014.

[ref15] Hohenberg P.; Kohn W. Inhomogeneous Electron Gas. Phys. Rev. 1964, 136, B864–B871. 10.1103/PhysRev.136.B864.

[ref16] Kohn W.; Sham L. J. Self-Consistent Equations Including Exchange and Correlation Effects. Phys. Rev. 1965, 140, A1133–A1138. 10.1103/PhysRev.140.A1133.

[ref17] Staemmler V. Method of Local Increments for the Calculation of Adsorption Energies of Atoms and Small Molecules on Solid Surfaces. 2. CO/MgO(001). J. Phys. Chem. A 2011, 115, 7153–7160. 10.1021/jp200047d. 21513315

[ref18] Kristyán S.; Pulay P. Can (semi)local density functional theory account for the London dispersion forces?. Chem. Phys. Lett. 1994, 229, 175–180. 10.1016/0009-2614(94)01027-7.

[ref19] Wu X.; Vargas M. C.; Nayak S.; Lotrich V.; Scoles G. Towards extending the applicability of density functional theory to weakly bound systems. J. Chem. Phys. 2001, 115, 8748–8757. 10.1063/1.1412004.

[ref20] Wesolowski T. A.; Morgantini P.-Y.; Weber J. Intermolecular interaction energies from the total energy bifunctional: A case study of carbazole complexes. J. Chem. Phys. 2002, 116, 6411–6421. 10.1063/1.1462613.

[ref21] Grimme S. Accurate description of van der Waals complexes by density functional theory including empirical corrections. J. Comput. Chem. 2004, 25, 1463–1473. 10.1002/jcc.20078. 15224390

[ref22] Kerber T.; Sierka M.; Sauer J. Application of semiempirical long-range dispersion corrections to periodic systems in density functional theory. J. Comput. Chem. 2008, 29, 2088–2097. 10.1002/jcc.21069. 18629806

[ref23] Grimme S. Semiempirical GGA-type density functional constructed with a long-range dispersion correction. J. Comput. Chem. 2006, 27, 1787–1799. 10.1002/jcc.20495. 16955487

[ref24] Grimme S.; Huenerbein R.; Ehrlich S. On the Importance of the Dispersion Energy for the Thermodynamic Stability of Molecules. ChemPhysChem 2011, 12, 1258–1261. 10.1002/cphc.201100127. 21445954

[ref25] Tkatchenko A.; Scheffler M. Accurate Molecular Van Der Waals Interactions from Ground-State Electron Density and Free-Atom Reference Data. Phys. Rev. Lett. 2009, 102, 73005 10.1103/PhysRevLett.102.073005. 19257665

[ref26] Hirata S.; Podeszwa R.; Tobita M.; Bartlett R. J. Coupled-cluster singles and doubles for extended systems. J. Chem. Phys. 2004, 120, 2581–2592. 10.1063/1.1637577. 15268402

[ref27] Marsman M.; Grüneis A.; Paier J.; Kresse G. Second-order Møller–Plesset perturbation theory applied to extended systems. I. Within the projector-augmented-wave formalism using a plane wave basis set. J. Chem. Phys. 2009, 130, 184103 10.1063/1.3126249. 19449904

[ref28] Müller C.; Paulus B. Wavefunction-based electron correlation methods for solids. Phys. Chem. Chem. Phys. 2012, 14, 7605 10.1039/c2cp24020c. 22373600

[ref29] Booth G. H.; Grüneis A.; Kresse G.; Alavi A. Towards an exact description of electronic wavefunctions in real solids. Nature 2013, 493, 365–370. 10.1038/nature11770. 23254929

[ref30] Yang J.; Hu W.; Usvyat D.; Matthews D.; Schütz M.; Chan G. K.-L. Ab initio determination of the crystalline benzene lattice energy to sub-kilojoule/mol accuracy. Science 2014, 345, 640–643. 10.1126/science.1254419. 25104379

[ref31] Lau B. T. G.; Knizia G.; Berkelbach T. C. Regional Embedding Enables High-Level Quantum Chemistry for Surf. Sci. J. Phys. Chem. Lett. 2021, 12, 1104–1109. 10.1021/acs.jpclett.0c03274. 33475362

[ref32] Chulhai D. V.; Goodpaster J. D. Projection-Based Correlated Wave Function in Density Functional Theory Embedding for Periodic Systems. J. Chem. Theory Comput. 2018, 14, 1928–1942. 10.1021/acs.jctc.7b01154. 29494155

[ref33] Ye H.-Z.; Berkelbach T. C. Correlation-Consistent Gaussian Basis Sets for Solids Made Simple. J. Chem. Theory Comput. 2022, 18, 1595–1606. 10.1021/acs.jctc.1c01245. 35192359

[ref34] Szabo A.; Ostlund N. S. Modern Quantum Chemistry: Introduction to Advanced Electronic Structure Theory, 1st ed.; Dover Publications, Inc.: Mineola, NY, 1996.

[ref35] Pacchioni G.; Cogliandro G.; Bagus P. S. Characterization of oxide surfaces by infrared spectroscopy of adsorbed carbon monoxide: A theoretical investigation of the frequency shift of CO on MgO and NiO. Surf. Sci. 1991, 255, 344–354. 10.1016/0039-6028(91)90691-K.

[ref36] Neyman K. M.; Rösch N. CO bonding and vibrational modes on a perfect MgO(001) surface: LCGTO-LDF model cluster investigation. Chem. Phys. 1992, 168, 267–280. 10.1016/0301-0104(92)87161-2.

[ref37] Ugliengo P.; Damin A. Are dispersive forces relevant for CO adsorption on the MgO(001) surface?. Chem. Phys. Lett. 2002, 366, 683–690. 10.1016/S0009-2614(02)01657-3.

[ref38] Nolan S. J.; Gillan M. J.; Alfè D.; Allan N. L.; Manby F. R. Calculation of properties of crystalline lithium hydride using correlated wave function theory. Phys. Rev. B 2009, 80, 165109 10.1103/PhysRevB.80.165109.

[ref39] Usvyat D.; Sadeghian K.; Maschio L.; Schütz M. Geometrical frustration of an argon monolayer adsorbed on the MgO (100) surface: An accurate periodic *ab initio* study. Phys. Rev. B 2012, 86, 45412 10.1103/PhysRevB.86.045412.

[ref40] Sun Q.; Chan G. K.-L. Quantum Embedding Theories. Acc. Chem. Res. 2016, 49, 2705–2712. 10.1021/acs.accounts.6b00356. 27993005

[ref41] Christlmaier E. M.; Kats D.; Alavi A.; Usvyat D. Full configuration interaction quantum Monte Carlo treatment of fragments embedded in a periodic mean field. J. Chem. Phys. 2022, 156, 154107 10.1063/5.0084040. 35459290

[ref42] Helgaker T.; Jørgensen P.; Olsen J. Molecular Electronic-Structure Theory; John Wiley & Sons, Inc.: Hoboken, NJ, 2014; pp 1–908.

[ref43] Knizia G.; Chan G. K.-L. Density Matrix Embedding: A Simple Alternative to Dynamical Mean-Field Theory. Phys. Rev. Lett. 2012, 109, 186404 10.1103/PhysRevLett.109.186404. 23215304

[ref44] Georges A.; Kotliar G.; Krauth W.; Rozenberg M. J. Dynamical Mean-Field Theory of Strongly Correlated Fermion Systems and The Limit of Infinite Dimensions. Rev. Mod. Phys. 1996, 68, 13–125. 10.1103/RevModPhys.68.13.

[ref45] Knizia G.; Chan G. K.-L. Density Matrix Embedding: A Strong-Coupling Quantum Embedding Theory. J. Chem. Theory Comput. 2013, 9, 1428–1432. 10.1021/ct301044e. 26587604

[ref46] Wouters S.; Jiménez-Hoyos C. A.; Sun Q.; Chan G. K.-L. A Practical Guide to Density Matrix Embedding Theory in Quantum Chemistry. J. Chem. Theory Comput. 2016, 12, 2706–2719. 10.1021/acs.jctc.6b00316. 27159268

[ref47] Zheng B.-X.; Chan G. K.-L. Ground-State Phase Diagram of The Square Lattice Hubbard Model from Density Matrix Embedding Theory. Phys. Rev. B 2016, 93, 035126 10.1103/PhysRevB.93.035126.

[ref48] Reinhard T. E.; Mordovina U.; Hubig C.; Kretchmer J. S.; Schollwöck U.; Appel H.; Sentef M. A.; Rubio A. Density-Matrix Embedding Theory Study of The One-Dimensional Hubbard-Holstein Model. J. Chem. Theory Comput. 2019, 15, 2221–2232. 10.1021/acs.jctc.8b01116. 30807149 PMC6674265

[ref49] Wouters S.; Jiménez-Hoyos C. A.; Chan G. K. L. Fragmentation; John Wiley & Sons, Inc.: Hoboken, NJ, 2017; Chapter 8, pp 227–243.

[ref50] Pham H. Q.; Bernales V.; Gagliardi L. Can Density Matrix Embedding Theory with The Complete Activate Space Self-Consistent Field Solver Describe Single and Double Bond Breaking in Molecular Systems?. J. Chem. Theory Comput. 2018, 14, 1960–1968. 10.1021/acs.jctc.7b01248. 29481744

[ref51] Nusspickel M.; Booth G. H. Systematic Improvability in Quantum Embedding for Real Materials. Phys. Rev. X 2022, 12, 11046 10.1103/PhysRevX.12.011046.

[ref52] Hermes M. R.; Gagliardi L. Multiconfigurational Self-Consistent Field Theory with Density Matrix Embedding: The Localized Active Space Self-Consistent Field Method. J. Chem. Theory Comput. 2019, 15, 972–986. 10.1021/acs.jctc.8b01009. 30620876

[ref53] Hermes M. R.; Pandharkar R.; Gagliardi L. Variational Localized Active Space Self-Consistent Field Method. J. Chem. Theory Comput. 2020, 16, 4923–4937. 10.1021/acs.jctc.0c00222. 32491849

[ref54] Faulstich F. M.; Kim R.; Cui Z.-H.; Wen Z.; Kin-Lic Chan G.; Lin L. Pure State v-Representability of Density Matrix Embedding Theory. J. Chem. Theory Comput. 2022, 18, 851–864. 10.1021/acs.jctc.1c01061. 35084855

[ref55] Peschel I.; Eisler V. Reduced Density Matrices and Entanglement Entropy in Free Lattice Models. J. Phys. A 2009, 42, 504003 10.1088/1751-8113/42/50/504003.

[ref56] Pham H. Q.; Hermes M. R.; Gagliardi L. Periodic Electronic Structure Calculations with The Density Matrix Embedding Theory. J. Chem. Theory Comput. 2020, 16, 130–140. 10.1021/acs.jctc.9b00939. 31815455

[ref57] Cui Z.-H.; Zhu T.; Chan G. K.-L. Efficient Implementation of Ab Initio Quantum Embedding in Periodic Systems: Density Matrix Embedding Theory. J. Chem. Theory Comput. 2020, 16, 119–129. 10.1021/acs.jctc.9b00933. 31815466

[ref58] Pham H. Q. pDMET: A Code for Periodic DMET Calculations, 2019; https://github.com/hungpham2017/pDMET (accessed Sept 29, 2021).

[ref59] Mitra A. pDMET: A Code for Periodic DMET Calculations, 2022; https://github.com/mitra054/pDMET (accessed Feb 24, 2022).

[ref60] Sun Q.; Berkelbach T. C.; Blunt N. S.; Booth G. H.; Guo S.; Li Z.; Liu J.; McClain J. D.; Sayfutyarova E. R.; Sharma S.; Wouters S.; Chan G. K.-L. PySCF: The Python-Based Simulations of Chemistry Framework. Wiley Interdiscip. Rev. Comput. 2018, 8, e1340 10.1002/wcms.1340.

[ref61] Sun Q.; Zhang X.; Banerjee S.; Bao P.; Barbry M.; Blunt N. S.; Bogdanov N. A.; Booth G. H.; Chen J.; Cui Z.-H.; Eriksen J. J.; Gao Y.; Guo S.; Hermann J.; Hermes M. R.; Koh K.; Koval P.; Lehtola S.; Li Z.; Liu J.; Mardirossian N.; McClain J. D.; Motta M.; Mussard B.; Pham H. Q.; Pulkin A.; Purwanto W.; Robinson P. J.; Ronca E.; Sayfutyarova E. R.; Scheurer M.; Schurkus H. F.; Smith J. E. T.; Sun C.; Sun S.-N.; Upadhyay S.; Wagner L. K.; Wang X.; White A.; Whitfield J. D.; Williamson M. J.; Wouters S.; Yang J.; Yu J. M.; Zhu T.; Berkelbach T. C.; Sharma S.; Sokolov A. Y.; Chan G. K.-L. Recent Developments in The PySCF Program Package. J. Chem. Phys. 2020, 153, 024109 10.1063/5.0006074. 32668948

[ref62] Mitra A.; Pham H. Q.; Pandharkar R.; Hermes M. R.; Gagliardi L. Excited States of Crystalline Point Defects with Multireference Density Matrix Embedding Theory. J. Phys. Chem. Lett. 2021, 12, 11688–11694. 10.1021/acs.jpclett.1c03229. 34843250

[ref63] Marzari N.; Vanderbilt D. Maximally Localized Generalized Wannier Functions for Composite Energy Bands. Phys. Rev. B 1997, 56, 12847–12865. 10.1103/PhysRevB.56.12847.

[ref64] Marzari N.; Mostofi A. A.; Yates J. R.; Souza I.; Vanderbilt D. Maximally Localized Wannier Functions: Theory and Applications. Rev. Mod. Phys. 2012, 84, 1419–1475. 10.1103/RevModPhys.84.1419.

[ref65] Pizzi G.; Vitale V.; Arita R.; Blügel S.; Freimuth F.; Géranton G.; Gibertini M.; Gresch D.; Johnson C.; Koretsune T.; Ibañez-Azpiroz J.; Lee H.; Lihm J.-M.; Marchand D.; Marrazzo A.; Mokrousov Y.; Mustafa J. I.; Nohara Y.; Nomura Y.; Paulatto L.; Poncé S.; Ponweiser T.; Qiao J.; Thöle F.; Tsirkin S. S.; Wierzbowska M.; Marzari N.; Vanderbilt D.; Souza I.; Mostofi A. A.; Yates J. R. Wannier90 as A Community Code: New Features and Applications. J. Phys.: Condens. Matter 2020, 32, 165902 10.1088/1361-648X/ab51ff. 31658458

[ref66] Pham H. Q. pyWannier90: A Python Interface for wannier90, 2019; https://github.com/hungpham2017/pyWannier90 (accessed Sept 29, 2021).

[ref67] Beebe N. H. F.; Linderberg J. Simplifications in the generation and transformation of two-electron integrals in molecular calculations. Int. J. Quantum Chem. 1977, 12, 683–705. 10.1002/qua.560120408.

[ref68] Vahtras O.; Almlöf J.; Feyereisen M. W. Integral approximations for LCAO-SCF calculations. Chem. Phys. Lett. 1993, 213, 514–518. 10.1016/0009-2614(93)89151-7.

[ref69] Koch H.; Sánchez de Merás A.; Pedersen T. B. Reduced scaling in electronic structure calculations using Cholesky decompositions. J. Chem. Phys. 2003, 118, 9481–9484. 10.1063/1.1578621.

[ref70] Røeggen I.; Wisløff-Nilssen E. On the Beebe-Linderberg two-electron integral approximation. Chem. Phys. Lett. 1986, 132, 154–160. 10.1016/0009-2614(86)80099-9.

[ref71] Goedecker S.; Teter M.; Hutter J. Separable Dual-Space Gaussian Pseudopotentials. Phys. Rev. B 1996, 54, 1703–1710. 10.1103/PhysRevB.54.1703. 9986014

[ref72] Hartwigsen C.; Goedecker S.; Hutter J. Relativistic separable dual-space Gaussian pseudopotentials from H to Rn. Phys. Rev. B 1998, 58, 3641–3662. 10.1103/PhysRevB.58.3641. 9986014

[ref73] Nygren M. A.; Pettersson L. G. M.; Barandiarán Z.; Seijo L. Bonding between CO and the MgO(001) surface: A modified picture. J. Chem. Phys. 1994, 100, 2010–2018. 10.1063/1.466553.

[ref74] Roos B. O.; Taylor P. R.; Sigbahn P. E. A Complete Active Space SCF Method (CASSCF) using A Density Matrix Formulated Super-CI Approach. Chem. Phys. 1980, 48, 157–173. 10.1016/0301-0104(80)80045-0.

[ref75] Siegbahn P. E. M.; Almlöf J.; Heiberg A.; Roos B. O. The Complete Active Space SCF (CASSCF) Method in A Newton–Raphson Formulation with Application to The HNO Molecule. J. Chem. Phys. 1981, 74, 2384–2396. 10.1063/1.441359.

[ref76] Siegbahn P.; Heiberg A.; Roos B.; Levy B. A Comparison of The Super-CI and The Newton-Raphson Scheme in The Complete Active Space SCF Method. Phys. Scr. 1980, 21, 323–327. 10.1088/0031-8949/21/3-4/014.

[ref77] Angeli C.; Cimiraglia R.; Evangelisti S.; Leininger T.; Malrieu J.-P. Introduction of n-electron Valence States for Multireference Perturbation Theory. J. Chem. Phys. 2001, 114, 10252–10264. 10.1063/1.1361246.

[ref78] Angeli C.; Borini S.; Cestari M.; Cimiraglia R. A Quasidegenerate Formulation of The Second Order N-Electron Valence State Perturbation Theory Approach. J. Chem. Phys. 2004, 121, 4043–4049. 10.1063/1.1778711. 15332949

[ref79] Angeli C.; Cimiraglia R.; Malrieu J.-P. N-electron Valence State Perturbation Theory: A Fast Implementation of The Strongly Contracted Variant. Chem. Phys. Lett. 2001, 350, 297–305. 10.1016/S0009-2614(01)01303-3.

[ref80] Angeli C.; Cimiraglia R.; Malrieu J.-P. N-electron Valence State Perturbation Theory: A Spinless Formulation and An Efficient Implementation of The Strongly Contracted and of The Partially Contracted Variants. J. Chem. Phys. 2002, 117, 9138–9153. 10.1063/1.1515317.

