# Supplementary material for: Periodic Density Matrix Embedding for CO Adsorption on the MgO(001) Surface
Source: J Phys Chem Lett. 2022 Aug 8;13(32):7483–9. doi: 10.1021/acs.jpclett.2c01915 (PMC9393885; doi:10.1021/acs.jpclett.2c01915)
Supplement: Supplementary file 1 — jz2c01915_si_001.pdf [file jz2c01915_si_001.pdf]

Supporting Information:

Supporting Information

Periodic Density Matrix Embedding for CO Adsorption on the MgO(001)Surface

Abhishek Mitra,[†] Matthew R. Hermes,[†] Minsik Cho,[‡] Valay Agarawal,[†] and Laura Gagliardi[*,¶,§]

†*Department of Chemistry, Chicago Center for Theoretical Chemistry, University of Chicago, Chicago, IL 60637, USA.*

‡*Department of Chemistry, Brown University, Providence, RI 02912, USA.*

¶*Department of Chemistry, Pritzker School of Molecular Engineering, James Franck Institute, Chicago Center for Theoretical Chemistry, University of Chicago, Chicago, IL 60637, USA.*

§*Argonne National Laboratory 9700 S. Cass Avenue Lemont, IL 60439*

E-mail: lgagliardi@uchicago.edu

Contents

S01 Mean Absolute Deviations (MADs) **S3**

S02 Adsorption energies **S3**

S03 Relative energies for C-Mg distances **S5**

S04 Memory Savings for DF integrals **S5**

S05 Sample Input and Output Files **S6**

S06 Total Energies **S7**

S0References **S9**

S01. Mean Absolute Deviations (MADs)

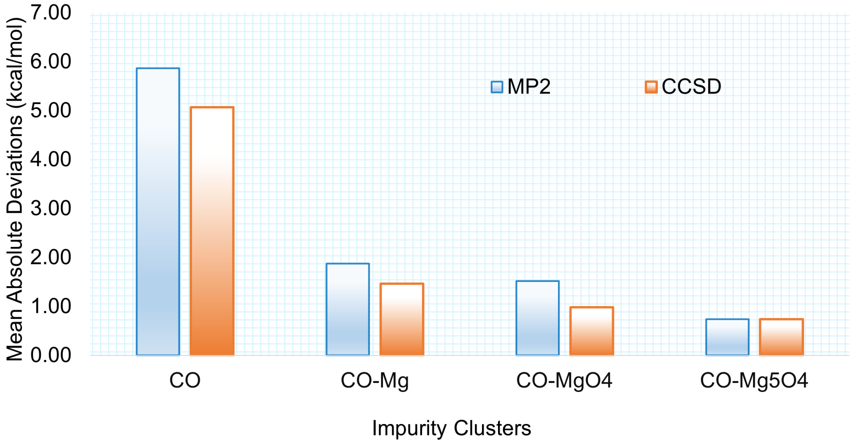

Figure S1: Mean Absolute Deviations (MADs) of DMET-MP2 (blue) and DMET-CCSD (saffron) from the non-embedded MP2 and CCSD references. The abscissa refers to the impurity clusters that use the high-level solvers.

Table S1: Mean Absolute Deviations (MADs) of DMET-MP2 (blue) and DMET-CCSD (saffron) from the non-embedded MP2 and CCSD references.

Method (Impurity)	Mean Absolute Deviations (kcal/mol)	
	DMET-MP2	DMET-CCSD
DMET(CO)	5.86	5.07
DMET(CO+Mg)	1.88	1.47
DMET(CO+MgO_4)	1.52	0.98
DMET(CO+Mg_5O_4)	0.74	0.74

S02. Adsorption energies

Table S2: Energy differences (in kcal/mole) between the equilibrium (2.479 Å)[S1] and separated (6.0 Å) geometries of the CO molecule on the MgO(001) surface using DMET with MP2 as the impurity solver compared to the MP2 non-embedding reference. "Reference" here indicates the non-embedded Γ-point MP2 calculation.

Method (Impurity)	Adsorption Energy (Deviation) in kcal/mol			
	DZVP	TZVP(CO)	TZVP(CO+Mg)	TZVP(CO+Mg_5O_4)
DMET(CO)	-3.45	-7.38	-4.24	-3.20
DMET(CO+Mg)	-7.52	-9.84	-12.20	-7.29
DMET(CO+MgO_4)	-7.15	-11.63	-8.93	-7.95
DMET(CO+Mg_5O_4)	-8.54	-13.52	-9.94	-9.36
reference	-8.67	-12.22	-10.89	-9.95

Table S3: Energy differences (in kcal/mole) between the equilibrium (2.479 Å) and separated (6.0 Å) geometries of the CO molecule on the MgO(001) surface obtained using DMET with CCSD the solvers compared to the CCSD non-embedding reference. "Reference" here indicates the non-embedded Γ-point CCSD calculation.

Method (Impurity)	Energy differences in kcal/mol			
	DZVP	TZVP(CO)	TZVP(CO+Mg)	TZVP(CO+Mg_5O_4)
DMET(CO)	-3.35	-7.63	-4.0	-2.82
DMET(CO+Mg)	-7.24	-9.86	-12.33	-7.45
DMET(CO+MgO_4)	-8.04	-11.64	-8.11	-7.93
DMET(CO+Mg_5O_4)	-6.69	-11.74	-9.36	-8.86
reference	-7.82	-11.08	-10.02	-9.18

S03. Relative energies for C-Mg distances

Table S4: Relative energies (in kcal/mol) obtained with respect to the equilibrium geometry using non-embedding MP2, DMET-MP2 with C, O and Mg atoms in the impurity cluster, DMET-MP2 with $CO+MgO_4$ in the impurity cluster and restricted Hartree-Fock. All calculations are performed using the DZVP basis set.

C-Mg distance (Å)	RHF (kcal/mol)	MP2 (kcal/mol)	DMET-MP2 (3) (kcal/mol)	DMET-MP2 (7) (kcal/mol)
2.0	14.19	9.37	12.53	10.12
2.479	0.00	0.00	0.00	0.00
3.0	-0.97	2.46	0.23	1.16
4.0	-0.38	6.68	5.72	5.09
5.0	-0.05	8.34	7.59	7.71
6.0	0.01	8.67	7.52	7.15

S04. Memory Savings for DF integrals

As reported in the manuscript, for a particular test case involving the $CO+Mg_5O_4$ fragment for the equilibrium geometry, we observe a 5-7 fold memory efficiency for our improved algorithm. We started by allocating 20 Gb of memory and increased the maximum memory for each calculation by 10 gigabytes (GB) and observed that our current implementation can perform total energy calculations using 30 GB whereas we need at least 200 GB of memory for the same calculation using the previous versions of the periodic DMET implementation.[S2] As discussed in the manuscript, this is because the new implementation requires storage of $O(N_{\text{aux}}N_{\text{imp}}^2)$ Cholesky vector elements whereas the previous implementation in Refs. S2 and S3 required storage of $O(N_{\text{imp}}^4)$ two-electron integrals. N_{imp} is the number of impurity fragment orbitals and N_{aux} is the number of auxiliary density-fitting orbitals. For large impurity fragments, $N_{\text{imp}}^2 \gg N_{\text{aux}}$ and the storage saving becomes significant.

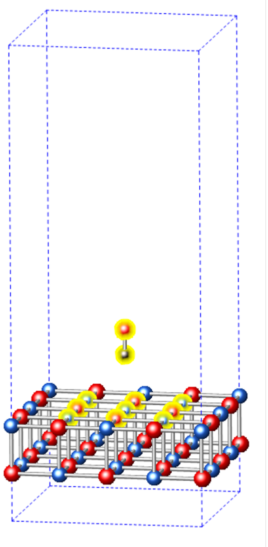

Memory	4 c-2 e	DF integrals
20G	✗	✗
30G	✗	✓
190G	✗	✓
200G	✓	✓

Figure S2: Total energies calculated using DMET-MP2 (highlighted atoms as fragment) using the DZVP basis set for different amounts of maximum memory provided to the supercomputers. DF integrals refer to our current density fitting implementation whereas 4c-2e refers to the previous algorithm. The maximum memory was increased in batches of 10 gigabytes. The absolute energies for both calculations were exact to $10^{-8} E_h$.

S05. Sample Input and Output Files

A sample python scrips to run a periodic MP2-DMET calculation titled "CO_MgO_2atoms.py" has been included in the folder Sample_python_scripts along with the obtained output. The input file in particular uses C and O atoms in the impurity region. The calculation is done using the TZVP basis sets on the CO+Mg_5O_4 atoms and DZVP on all others. The MLWFs corresponding to this system are also included for reference in Sample_calculation/MLWFs. In lines 229-233, the output file prints out the orbitals (MLWFs) which correspond to these particular atoms. The same format can be used to run a CCSD-DMET calculation by only replacing pdmet = dmet.pDMET(cell, kmf, w90, solver='MP2') with pdmet = dmet.pDMET(cell, kmf, w90, solver='CCSD'). The DMET output section starts from Line 219 in the file 'CO_MgO_2atoms.out.py'. In line 247, the total energy per unit cell is printed out.

S06. Total Energies

Table S5: Total energy (in Hartree) for the equilibrium (2.479 Å)[S1] geometry labelled as Eq. and separated (6.0 Å) geometry labelled as Sep. of the CO molecule on the MgO(001) surface using DMET with MP2 as the impurity solver compared to the MP2 non-embedding reference. All calculations are done using the DZVP basis sets. "Emb. orbs" refer to the number of embedding orbitals in the calculations. "Reference" here indicates the non-embedded Γ-point MP2/CCSD calculation.

Impurity cluster	Emb. orbs.	CCSD		MP2	
		Eq.	Sep.	Eq.	Sep.
CO	52	-1271.13817	-1271.13282	-1271.14505	-1271.13955
CO+Mg	74	-1271.16198	-1271.15045	-1271.16798	-1271.15599
CO+MgO_4	178	-1271.97229	-1271.95947	-1272.01358	-1272.00218
CO+Mg_5O_4	266	-1272.12741	-1272.11674	-1272.16717	-1272.15356
reference	458	-1274.68331	-1274.67084	-1274.86392	-1274.85011

Table S6: Total energies (in Hartree) obtained using non-embedding MP2, DMET-MP2 with C, O and Mg atoms in the impurity cluster, DMET-MP2 with CO+MgO_4 in the impurity cluster and restricted Hartree-Fock. All calculations are performed using the DZVP basis set.

C-Mg distance	RHF	MP2	DMET-MP2 (3)	DMET-MP2 (7)
2.0	-1270.82114	-1274.84900	-1271.14801	-1271.99745
2.479	-1270.84375	-1274.86392	-1271.16798	-1272.01358
3.0	-1270.84530	-1274.86000	-1271.16761	-1272.01173
4.0	-1270.84435	-1274.85328	-1271.15886	-1272.00547
5.0	-1270.84384	-1274.85063	-1271.15588	-1272.00129
6.0	-1270.84374	-1274.85011	-1271.15599	-1272.00218

Table S7: Total energy (in Hartree) for the equilibrium (2.479 Å)[S1] geometry labelled as Eq. and separated (6.0 Å) geometry labelled as Sep. of the CO molecule on the MgO(001) surface using DMET with MP2 as the impurity solver compared to the MP2 non-embedding reference. All calculations are done using the TZVP basis sets on the CO atoms and DZVP on all others. "Emb. orbs" refer to the number of embedding orbitals in the calculations. "Reference" here indicates the non-embedded Γ-point MP2/CCSD calculation.

Impurity cluster	Emb. orbs.	MP2		CCSD	
		Eq	Sep	Eq	Sep
CO	58	-1271.16242	-1271.15066	-1271.15462	-1271.14245
CO+Mg	80	-1271.18265	-1271.16697	-1271.17528	-1271.15957
CO+MgO_4	184	-1272.03039	-1272.01185	-1271.98821	-1271.96966
CO+Mg_5O_4	272	-1272.17867	-1272.15713	-1272.13698	-1272.11827
reference	466	-1274.89242	-1274.87294	-1274.71045	-1274.69278

Table S8: Total energy (in Hartree) for the equilibrium (2.479 Å)[S1] geometry labelled as Eq. and separated (6.0 Å) geometry labelled as Sep. of the CO molecule on the MgO(001) surface using DMET with MP2 as the impurity solver compared to the MP2 non-embedding reference. All calculations are done using the TZVP basis sets on the CO+Mg atoms and DZVP on all others. "Emb. orbs" refer to the number of embedding orbitals in the calculations. "Reference" here indicates the non-embedded Γ-point MP2/CCSD calculation.

Impurity cluster	Emb. orbs.	MP2		CCSD	
		Eq	Sep	Eq	Sep
CO	68	-1271.17475	-1271.16799	-1271.16714	-1271.16070
CO+Mg	96	-1271.21887	-1271.19944	-1271.21329	-1271.19364
CO+MgO_4	200	-1272.06522	-1272.05100	-1272.02310	-1272.01018
CO+Mg_5O_4	288	-1272.26449	-1272.24865	-1272.22296	-1272.20831
reference	470	-1274.91735	-1274.89999	-1274.73419	-1274.71822

Table S9: Total energy (in Hartree) for the equilibrium (2.479 Å)[S1] geometry labelled as Eq. and separated (6.0 Å) geometry labelled as Sep. of the CO molecule on the MgO(001) surface using DMET with MP2 as the impurity solver compared to the MP2 non-embedding reference. All calculations are done using the TZVP basis sets on the CO+Mg_5O_4 atoms and DZVP on all others. "Emb. orbs" refer to the number of embedding orbitals in the calculations. "Reference" here indicates the non-embedded Γ-point MP2/CCSD calculation.

Impurity cluster	Emb. orbs	MP2		CCSD	
		Eq	Sep	Eq	Sep
CO	66	-1271.21659	-1271.21149	-1271.20897	-1271.20448
CO+Mg	94	-1271.25151	-1271.23989	-1271.24530	-1271.23343
CO+MgO_4	200	-1272.10950	-1272.09682	-1272.06697	-1272.05434
CO+Mg_5O_4	306	-1272.41705	-1272.40264	-1272.37131	-1272.35719
reference	502	-1275.09323	-1275.07737	-1274.90088	-1274.88624

References

(S1) Boese, A. D.; Sauer, J. Accurate adsorption energies of small molecules on oxide surfaces: CO-MgO(001). *Phys. Chem. Chem. Phys* **2013**, *15*, 16481–16493.

(S2) Pham, H. Q.; Hermes, M. R.; Gagliardi, L. Periodic Electronic Structure Calculations with The Density Matrix Embedding Theory. *J. Chem. Theory Comput.* **2020**, *16*, 130–140.

(S3) Mitra, A.; Pham, H. Q.; Pandharkar, R.; Hermes, M. R.; Gagliardi, L. Excited States of Crystalline Point Defects with Multireference Density Matrix Embedding Theory. *J. Phys. Chem. Lett* **2021**, *12*, 11688–11694.
