# Supplementary material for: Periodic Density Matrix Embedding for CO Adsorption on the MgO(001) Surface
Source: J Phys Chem Lett. 2022 Aug 8;13(32):7483–9. doi: 10.1021/acs.jpclett.2c01915 (PMC9393885; doi:10.1021/acs.jpclett.2c01915)
Supplement: Supplementary file 2 — jz2c01915_si_002.pdf [file jz2c01915_si_002.pdf]

jz-2022-019153.R1

Name: Peer Review Information for "Periodic Density Matrix Embedding for CO Adsorption on the MgO(001)Surface"

First Round of Reviewer Comments

Reviewer: 1

Comments to the Author

Mitra et al. present a benchmark of their Periodic Density Matrix Embedding implementation on a well-known, prototypical system for the study of surface adsorption, namely CO@MgO.

The details of the method are presented and the results are properly benchmarked against full periodic calculations, showing internal consistency and documenting the cluster size needed for a converged result in this particular case.

I believe that the work is sound and suitable for publication, but at the same time I find the paper quite technical and I do not think that the findings are potentially of interest to a broad community, also considered that the system itself is more than well known (which is good for benchmarks). I therefore consider the article more suitable for other journals such as. JPCC or JCTC.

I have a number of more detailed comments.

- The use of pseudopotentials is mentioned only in passing. Do the authors believe that their findings would change in the case of an all-electron calculation? Why or why not?

- The Density Fitting equations (4,5,6) are rather standard molecular density fitting. I wonder whether there is a truly added value in reporting them explicitly rather than just citing the relevant references.

- I believe that reformulation of the MP2 or CCSD equations so to never reconstruct the full 4-index ERI is common practice in Density Fitting algorithms, so I might be missing the novelty of the author's approach.

- It is mentioned that calculations are performed on a 32-core processor, but it is not clear whether the code is parallelized, and according to what scheme (memory shared, replicated, ...). This is critical in understanding the data reported on memory usage.

-The memory requirements are reported in a rather empirical way, mentioning which calculation manages to 'get through' if a certain amount of Gigabytes is granted (e.g. Figure S2). It would be much more interesting to know the actual memory occupation for each of the quantities involved.

- The authors use the term "performance" in a rather broad way, referring from time to time to either quality of the result or need for memory resources. On the other hand the time required for a calculation is never mentioned.

Author's Response to Peer Review Comments:

Manuscript ID: jz-2022-019153

Responses to Review: Periodic Density Matrix Embedding for CO Adsorption on the MgO(001)Surface

Abhishek Mitra, Matthew R. Hermes, Minsik Cho, Valay Agarwal, and Laura Gagliardi

Replies to reviewer comments and list of changes

We appreciate the reviewer's careful reading and thank them for their insightful comments. The point-to-point responses are listed below. The reviewer comments are listed in blue, our responses in black, and changes in the Letter in red.

Reviewer 1

Mitra *et al.* present a benchmark of their Periodic Density Matrix Embedding implementation on a well-known, prototypical system for the study of surface adsorption, namely CO@MgO. The details of the method are presented and the results are properly benchmarked against full periodic calculations, showing internal consistency and documenting the cluster size needed for a converged result in this particular case. I believe that the work is sound and suitable for publication, but at the same time I find the paper quite technical and I do not think that the findings are potentially of interest to a broad community, also considered that the system itself is more than well known (which is good for benchmarks). I therefore consider the article more suitable for other journals such as. JPCC or JCTC.

Response:

We thank the reviewer for their summary and comments.

In the following we explain why we think that this paper may be of interest to the JPC Letters audience. It is true that the adsorption of CO on a MgO surface is a well known phenomenon. However, it is a prototypical example of chemical situations in which van der Waals (vdW) interactions play a major role and quantum chemical modeling of these interaction may be challenging. On one hand, many local and semi-local Kohn-Sham density functionals are often inaccurate,[1–4] thereby requiring an extensive testing of DFT functionals and the incorporation of dispersion corrections. Size-consistent correlated wave-function based *ab initio* methods, on the other hand, can accurately describe vDW interactions,[1] but are expensive. In this study we show that quantum embedding methods based on periodic DMET can accurately describe the binding of CO to a MgO surface when compared to correlated wave-function based *ab initio* methods at a significantly cheaper cost, which makes these methods appealing for future applications. Thus, given the potential of wave-function based methods for extended systems and phenomena like catalysis, this paper will interest the JPC Letters audience of theorists, modelers and experimentalits.

(1) The use of pseudopotentials is mentioned only in passing. Do the authors believe that their findings would change in the case of an all-electron calculation? Why or why not?

Response:

The findings should not change since the adsorption energy primarily depends on valence electrons and orbitals and the pDMET algorithm is agnostic to the choice of basis set and model Hamiltonian. In this respect, pDMET should be considered in the same way other electronic structure methods.

We have made the following changes in Page 6 so that this point is clear to the audience.

Our approach is agnostic to whether we use pseudopotentials or an all-electron basis sets since surface adsorption primarily depends on valence electrons and orbitals.

(2) The density fitting equations (4,5,6) are rather standard molecular density fitting. I

wonder whether there is a truly added value in reporting them explicitly rather than just citing the relevant references.

(3) I believe that reformulation of the MP2 or CCSD equations so to never reconstruct the full 4-index ERI is common practice in Density Fitting algorithms, so I might be missing the novelty of the author's approach.

Response:

We agree with the reviewer that the density fitting equations are standard.

However, they are needed for the discussion following eq (6) (i.e., the paragraph beginning with *"In the previous implementations of periodic DMET ..."* on page 6). The citations to the relevant references are included.

The reviewer is correct that the algebraic substitution of the density-fitting equations into the programmable equations is standard practice. The novelty here is that previous implementations of periodic DMET did not fully exploit this practice because the Cholesky vectors were only directly visible to the whole-system, low-level Hartree–Fock layer. In the current implementation, the Cholesky vectors are used directly in both layers of the calculation. Equations 4, 5 and 6 explain this point, together with the description of their use in the paragraph beginning with *"In the previous implementations of periodic DMET ..."* on page 6. The previous implementations in Refs. 5 and 6 required storage of $O(N_{\text{imp}}^4)$ two-electron integrals and passed these integrals to the high-level solvers. The strategy implemented here requires storage of only the $O(N_{\text{aux}}N_{\text{imp}}^2)$ decomposed intermediates.

(4) It is mentioned that calculations are performed on a 32-core processor, but it is not clear whether the code is parallelized, and according to what scheme (memory shared, replicated, ...). This is critical in understanding the data reported on memory usage.

Response: Our calculations use multithreading (i.e., shared memory) only and no multiprocessing. As the reviewer recommends, we clarify this point by making the following changes in Page 11.

All the calculations use multithreading (*i.e.*, shared memory) only; there is no multiprocess-

ing.

(5) The memory requirements are reported in a rather empirical way, mentioning which calculation manages to 'get through' if a certain amount of Gigabytes is granted (e.g. Figure S2). It would be much more interesting to know the actual memory occupation for each of the quantities involved.

Response: In the paragraph "In the previous implementations ..." in Page 6, the memory occupation for Figure S2 has been discussed. In short, the objective of the paragraph is to show that the formal memory cost scaling of our approach is $O(N_{\text{aux}}N_{\text{imp}}^2)$as compared to $\mathcal{O}(N_{\text{imp}}^4)$. Using the example as in Figure S2, we show that in practice, this scaling is favorable for our applications. To make Section S04 (and therefore Figure S2) on Page 5 of the SI self contained, we add the following lines in the SI.

As discussed in the manuscript, this is because the new implementation requires storage of $O(N_{\text{aux}}N_{\text{imp}}^2)$ Cholesky vector elements whereas the previous implementation in Refs. 5 and 6 required storage of $O(N_{\text{imp}}^4)$ two-electron integrals. N_{imp} is the number of impurity fragment orbitals and N_{aux} is the number of auxiliary density-fitting orbitals. For large impurity fragments, $N_{\text{imp}}^2 \gg N_{\text{aux}}$ and the storage saving becomes significant.

(6) The authors use the term "performance" in a rather broad way, referring from time to time to either quality of the result or need for memory resources. On the other hand, the time required for a calculation is never mentioned.

Response:

The DF integrals implementation does not provide a formal speedup in terms of time required for a calculation. The only advantage it provides is a reduced memory requirement as has been mentioned in the manuscript. To highlight this point, we have made the following changes on Page 11 so that the effect of our new algorithm on the time taken for a calculation is understood. The time taken by the particular examples is also mentioned.

The current algorithm does not involve a speedup in terms of time required for a particular calculation. In this specific example, the 4c-2e calculation requires a wall-time of two hours

and twenty one minutes whereas the DF integrals calculation requires a wall-time of one hour and fifty five minutes.

The formal cost scaling due to quantum embedding has been discussed in Page 11 in the second to last paragraph of the manuscript.

The reviewer expresses concern over the use of the word "performance" in specific cases. We have reworded two occurrences (out of three) of the word "performance" and replaced them with the specific property involved. The reason for including the only occurrence of the word is explained below.

We have replaced the sentence on Page 9 as:

~~The performance of DMET, on the other hand, is encouraging.~~ DMET on the other hand reproduces the binding energy (to within 1.5 kcal/mol) that is predicted by the reference.

We have reworded an occurrence in Page 12 as:

We have investigated ~~the performance of~~ two widely used quantum chemical solvers, CCSD and MP2, as high-level methods.

We have however retained the use of the word "performance" in the Introduction section of Page 3 (*Modeling the adsorption of ...*) since this is a general statement.

References

(1) Staemmler, V. Method of Local Increments for the Calculation of Adsorption Energies of Atoms and Small Molecules on Solid Surfaces. 2. CO/MgO(001). *J. Phys. Chem. A* **2011**, *115*, 7153–7160.

(2) Kristyán, S.; Pulay, P. Can (semi)local density functional theory account for the London dispersion forces? *Chem. Phys. Lett* **1994**, *229*, 175–180.

(3) Wu, X.; Vargas, M. C.; Nayak, S.; Lotrich, V.; Scoles, G. Towards extending the ap-

plicability of density functional theory to weakly bound systems. *J. Phys. Chem.* **2001**, *115*, 8748–8757.

(4) Wesolowski, T. A.; Morgantini, P.-Y.; Weber, J. Intermolecular interaction energies from the total energy bifunctional: A case study of carbazole complexes. *J. Phys. Chem.* **2002**, *116*, 6411–6421.

(5) Pham, H. Q.; Hermes, M. R.; Gagliardi, L. Periodic Electronic Structure Calculations with The Density Matrix Embedding Theory. *J. Chem. Theory Comput.* **2020**, *16*, 130–140.

(6) Mitra, A.; Pham, H. Q.; Pandharkar, R.; Hermes, M. R.; Gagliardi, L. Excited States of Crystalline Point Defects with Multireference Density Matrix Embedding Theory. *J. Phys. Chem. Lett* **2021**, *12*, 11688–11694.
